# Conjugation Length Effect on TPA-Based Optical Limiting Performance of a Series of Ladder-Type Chromophores

**DOI:** 10.3390/ma10010070

**Published:** 2017-01-16

**Authors:** Yujin Zhang, Wei Hu, Liyun Zhao, Jiancai Leng, Hong Ma

**Affiliations:** 1 School of Science, Qilu University of Technology, Jinan 250353, China; zhangyujin312@163.com (Y.Z.); liyunzhao_mm@163.com (L.Z.); 2 Hefei National Laboratory for Physical Sciences at the Microscale, iChEM (Collaborative Innovation Center of Chemistry for Energy Materials), School of Chemistry and Materials Science, University of Science and Technology of China, Hefei 230026, China; weihukth@gmail.com; 3 School of Physics and Electronics, Shandong Normal University, Jinan 250014, China

**Keywords:** two-photon absorption, optical limiting, rate equations-field intensity equation

## Abstract

Nonlinear optical properties of a series of newly-synthesized ladder-type chromophores containing oligo-*p*-phenylene moiety with different π-conjugated lengths were theoretically studied by numerically solving the rate equations and the field intensity equation with an iterative predictor-corrector finite-difference time-domain technique. Ab initio calculation results show that the compounds can be described by the three-level model. Based on the two-photon absorption mechanism, highly efficient optical limiting performances are demonstrated in the chromophores, which strongly depend on the π-conjugated length of the molecule. Special attention has been paid to the dynamical two-photon absorption, indicating that the parameter of the medium can affect the dynamical two-photon absorption cross section. Our numerical results agree well with the experimental measurements. It reveals that the increase in the π-conjugated length of ladder-type oligo-*p*-phenylene for these chromophores leads to enhanced nonlinear optical absorption. The results also provide a method to modulate the optical limiting and dynamical two-photon absorption of the compounds by changing the molecular density and thickness of the absorber.

## 1. Introduction

In the modern laser technology, the protection of delicate sensors and human eyes from damage of intense laser radiation is in strong demand [1,2,3]. Under this context, devices with optical limiting (OL) abilities have attracted extensive attentions [4,5]. An excellent optical limiter shows high intensity transmission for relatively weak incident lasers, while it is opaque to intense lasers [6,7]. Such materials usually behave like a shutter responding on an ultrafast timescale. There are several mechanisms that can be used to achieve OL, among which two-photon absorption (TPA) is one of the most effective [8,9]. Thus, looking for optical limiters with strong TPA is a hot research topic in the field of optics [10,11].

During the last decade, a number of organic chromophores have been experimentally synthesized and theoretically designed as two-photon active materials that can be used as efficient optical limiters [12,13,14]. Due to the unique planer and rigid π-conjugation structure, fluorene-based chromophores are demonstrated to be excellent candidates for two-photon absorption and optical limiting devices [15,16,17]. Furthermore, ladder-type oligo-*p*-phenylene, which consists of several “linearly overlapping” fluorenes may act as a good building block of two-photon absorber or optical limiter due to its enhanced π-conjugation. However, most investigations on ladder-type oligo-*p*-phenylene are limited to its emission properties, which hinders its potential applications in the field of nonlinear optical absorption [18,19,20,21]. Recently, Zheng et al. reported the optical properties of a series of ladder-type oligo-*p*-phenylene-contained chromophores with various π-conjugation lengths (Mol-1, Mol-2, Mol-3, and Mol-4), showing that an increase in the π-conjugation length of these compounds leads to an enhancement in OL performance and an increase in the TPA cross section [22]. Even though the compounds exhibit prominent prospects as promising optical limiters experimentally, theoretical understanding on the mechanism of the TPA-based OL of the compounds are still rarely explored. Moreover, systematic theoretical investigations on the conjugation length effect on nonlinear optical properties for the given materials are insufficient. Moreover, molecular optical properties depend strongly on the interaction between the laser and the medium [23,24], while the dependence of parameters of pulse and the absorber on the OL performances of these compounds has not been discussed to date.

In this paper, ab initio calculations are firstly employed to simplify the compounds with a few energy levels. We then consider the interaction between laser and molecules by numerically solving the field intensity equation together with rate equations in a nanosecond time domain with the iterative predictor-corrector finite-difference time-domain (FDTD) method [25]. The propagation process of the laser in the medium and the OL behaviors of these compounds are investigated, and the dynamical TPA cross sections of these compounds are obtained. Both the conjugation length effect on the TPA-based optical limiting performance of these compounds and the dependence of the parameters of the absorber on the dynamical TPA cross sections are analyzed. The research results provide guidance for designing more excellent optical limiters.

## 2. Theoretical Methods and Computational Details

### 2.1. Ab Initio Calculations

**Figure 1:**
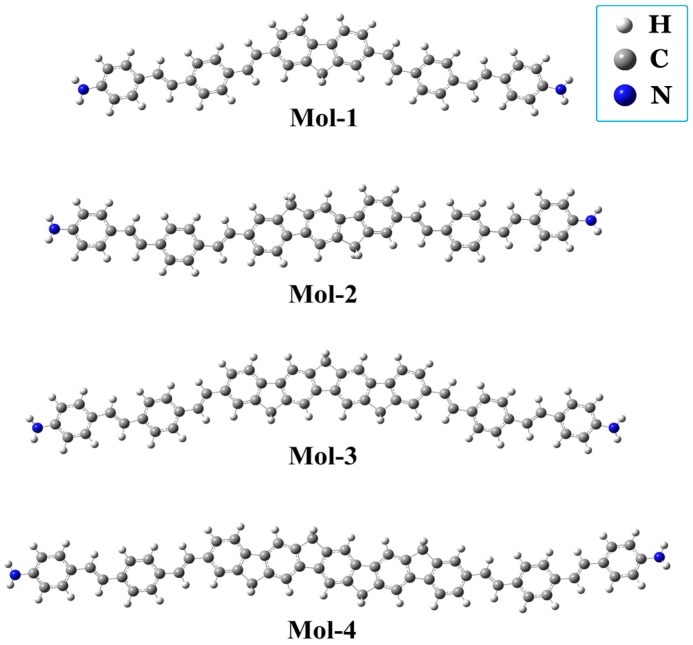
Molecular structures of Mol-1, Mol-2, Mol-3, and Mol-4.

**Figure 2:**
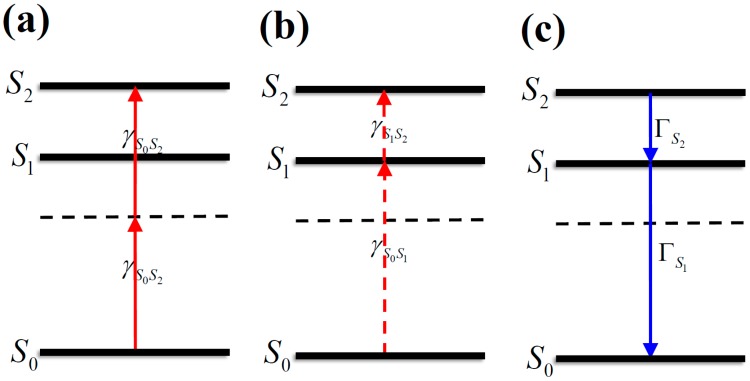
The transition scheme of a three-energy-level system: (**a**) one-step TPA; (**b**) two-step TPA; and (**c**) relaxation of the excited states.

Structures of the studied molecules are shown in Figure 1a. It can be seen that Mol-1, Mol-2, Mol-3, and Mol-4 are all π-conjugation symmetrical molecules. Ab initio calculations of these molecules, including geometry optimization and electronic structure calculations, are based on the time-dependent hybrid density functional theory (TDDFT)/Becke’s three-parametrized Lee–Yang–Parr (B3LYP) exchange functional level with the basis set of 6-31G(d) in the Dalton2013 program [26]. The calculated excitation energy, the corresponding wavelength, the oscillator strength, and the dipole moment of the first five excited states of Mol-1, Mol-2, Mol-3, and Mol-4 in gas phase are listed in Table 1. The results indicate that the first excited states of these chromophores have the maximum oscillator strength (δ_op_), and the dipole moments (μ_1*n*_) between the first excited states and the second excited states are the largest. Thus, when the interaction between laser and the molecule is dealt with, the molecules can be well described by a three-level model (as shown in Figure 2) in the low energy region, where *S*_0_, *S*_1_, and *S*_2_ are respectively the ground, first excited, and second excited states. The excitation energies of the *S*_1_ and *S*_2_ states are *E*_10_ = 2.51 eV and *E*_20_ = 2.83 eV for Mol-1, *E*_10_ = 2.53 eV and *E*_20_ = 2.83 eV for Mol-2, *E*_10_ = 2.53 eV and *E*_20_ = 2.81 eV for Mol-3, and *E*_10_ = 2.55 eV and *E*_20_ = 2.81 eV for Mol-4, respectively. It should be noted that, in the experiment, the linear absorption peaks of the compounds in different solvents are in the range of 420–450 nm. In comparison with the experimental values, our calculated results (486–494 nm) are much larger. This quantitative discrepancy may results from the fact that our ab initio calculation is based on the electronic structure of a single molecule in gas phase. Thus, the vibrational contribution, the interaction between molecules, and the solvent effect are not considered. The transition dipole moments among the states are μ_01_ = 7.54 a.u. and μ_12_ = 14.38 a.u. for Mol-1, μ_01_ = 8.12 a.u. and μ_12_ = 15.97 a.u. for Mol-2, μ_01_ = 8.46 a.u. and μ_12_ = 15.49 a.u. for Mol-3, and μ_01_ = 9.03 a.u. and μ_12_ = 15.63 a.u. for Mol-4, respectively. The one-photon transition between *S*_0_ and *S*_2_ is nearly dipole forbidden. Moreover, due to the molecular inversion symmetry property, the permanent dipole moments of these states are nearly equal to 0.

### 2.2. Rate Equations for a Three-Level System

Under the consideration of relaxation effect, the density matrix equation that describes the population of a certain energy level can be written as

(1) $$\begin{array}{ll} \frac{\partial }{\partial t}\rho_{mn}=-\frac{i}{\hbar }{\left[\hat{Η},\;\hat{\rho }\right]}_{mn}-\gamma_{mn}\rho_{mn} & m\neq n\\ \frac{\partial }{\partial t}\rho_{nn}=-\frac{i}{\hbar }{\left[\hat{Η},\;\hat{\rho }\right]}_{nn}+\sum_{E_{m}>E_{n}}\Gamma_{nm}\rho_{mm}+\sum_{E_{m}<E_{n}}\Gamma_{mn}\rho_{nn} \end{array}$$

where Γ*_mn_* is the decay rate of ρ*_nn_*, γ*_mn_* is the decay rate of ρ*_mn_*.

Based on the rotating wave approximation (RWA) and the adiabatic approximation ρ*’_mn_*(*t*) = 0, the density matrix equation for a three-level system (including states *S*_0_, *S*_1_ and *S*_2_) can be reduced to the following rate equations:

(2) $$\begin{array}{l} \frac{\partial }{\partial t}\rho_{S_{0}}=-\gamma_{S_{0}S_{1}}(\rho_{S_{0}}-\rho_{S_{1}})-\gamma_{S_{0}S_{2}}(\rho_{S_{0}}-\rho_{S_{2}})+\Gamma_{S_{1}}\rho_{S_{1}}\\ \frac{\partial }{\partial t}\rho_{S_{1}}=\gamma_{S_{0}S_{1}}(\rho_{S_{0}}-\rho_{S_{1}})-\gamma_{S_{1}S_{2}}(\rho_{S_{1}}-\rho_{S_{2}})-\Gamma_{S_{1}}\rho_{S_{1}}+\Gamma_{S_{2}}\rho_{S_{2}}\\ \frac{\partial }{\partial t}\rho_{S_{2}}=\gamma_{S_{0}S_{2}}(\rho_{S_{0}}-\rho_{S_{2}})+\gamma_{S_{1}S_{2}}(\rho_{S_{1}}-\rho_{S_{2}})-\Gamma_{S_{2}}\rho_{S_{2}} \end{array}$$

where ρ*_Sn_* represents the population of the state *S_n_*, and Γ*_S__n_* is the decay rate of the state *S_n_*. γ*_S__m__S__n_* is the one-photon transition rate between the states *S_m_* and *S_n_*, which can be expressed by the corresponding one-photon absorption (OPA) cross sections σ*_S__m__S__n_* and the dipole moments ***d****_S__m__S__n_* under RWA:

(3) $$\begin{array}{l} \gamma_{S_{0}S_{1}}(t)=\frac{{\left|d_{S_{0}S_{1}}\right|}^{2}I(t)}{\hbar^{2}c\varepsilon_{0}}\frac{\Gamma }{\Omega_{S_{0}S_{1}}^{2}+\Gamma^{2}}=\frac{\sigma_{S_{0}S_{1}}I(t)}{\hbar \omega }\frac{\Gamma^{2}}{\Omega_{S_{0}S_{1}}^{2}+\Gamma^{2}}\\ \gamma_{S_{1}S_{2}}(t)=\frac{{\left|d_{S_{1}S_{2}}\right|}^{2}I(t)}{\hbar^{2}c\varepsilon_{0}}\frac{\Gamma }{\Omega_{S_{1}S_{2}}^{2}+\Gamma^{2}}=\frac{\sigma_{S_{1}S_{2}}I(t)}{\hbar \omega }\frac{\Gamma^{2}}{\Omega_{S_{1}S_{2}}^{2}+\Gamma^{2}}\\ \Omega_{S_{0}S_{1}}=\omega -\omega_{S_{0}S_{1}}\;,\;\Omega_{S_{1}S_{2}}=\omega -\omega_{S_{1}S_{2}} \end{array}$$

where Ω*_S__m__S__n_* donates the detuning of light frequency ω from the resonant frequency ω*_S__m__S__n_* between the *S_m_* state and the *S_n_* state. *I*(*t*) = *c*ε_0_|*F*(*t*)|^2^/2 is the instantaneous intensity of the field, *F*(*t*) is the peak amplitude of the input electric field, and Γ is the homogeneous broadening of the spectral line. In this work, *ħ*Γ*_mn_* = *ħ*Γ = 0.1 eV is chosen for all transitions [27]. The two-photon transition rate γ*_S_0___S_2__* is similarly defined as

(4) $$\gamma_{S_{0}S_{2}}=\frac{\sigma_{S_{0}S_{2}}I^{2}(t)}{2\hbar \omega }\frac{\Gamma^{2}}{{\left(2\omega -\omega_{S_{0}S_{2}}\right)}^{2}+\Gamma^{2}}$$

where σ*_S_0___S_2__* denotes the TPA cross section.

It should be noted that the total populations of energy levels are normalized to one:

(5) $$\sum_{n=0}^{2}\rho_{S_{n}}=1$$

### 2.3. Field Intensity Equation

In the case of the nanosecond pulse propagating along the *z* axis through a three-level system, where the role of self-focusing and defocusing is small, one can ignore the change in refraction and transverse of the field. The absorption of the field can thus be described by the field intensity equation:

(6) $$\left(\frac{\partial }{\partial z}+\frac{1}{c}\frac{\partial }{\partial t})I(t)=-N[\sigma^{\left(1\right)}I(t)+\sigma^{\left(2\right)}I^{2}(t)\right]$$

where the total OPA cross section σ^(1)^ and TPA cross section σ^(2)^ are given by

(7) $$\sigma^{\left(1\right)}=\sigma_{S_{0}S_{1}}(\rho_{S_{0}}-\rho_{S_{1}})+\sigma_{S_{1}S_{2}}(\rho_{S_{1}}-\rho_{S_{2}}),\;\sigma^{\left(2\right)}=\sigma_{S_{0}S_{2}}(\rho_{S_{0}}-\rho_{S_{2}})$$

### 2.4. Dynamical TPA Cross Section

Assume that the TPA coefficient depends strongly on the input intensity *I*_0_ [28], namely,

(8) $$\beta =\beta_{0}-\xi I_{0}$$

where β_0_ is the inherent TPA coefficient that relates only with the molecule itself, and ξ is a constant related with the materials. The inverse intensity transmission *1/T(z*) is a quadratic function of the initial input field intensity *I*_0_:

(9) $$\frac{1}{T(z)}=\frac{I_{0}}{I(z)}=\exp(\alpha z)+\frac{\left[\exp(\alpha z)-1\right]\beta_{0}}{\alpha }I_{0}-\frac{\left[\exp(\alpha z)-1\right]\xi }{\alpha }I_{0}^{2}$$

The absorption coefficients can be determined by mathematically fitting Equation (9) using Origin software. Finally, one can obtain the dynamical TPA cross section σ_tp_ by

(10) $$hv\beta =\sigma_{\text{tp}}N$$

where *hv* is the incident photon energy, and *N* is the molecular density of the medium.

### 2.5. Computational Details

The molecule is assumed to be in its ground state *S*_0_ before the excitation laser pulse is switched on, namely, ρ_00_(*t* = 0) = 1 and ρ_11_(*t* = 0) = ρ_22_(*t* = 0) = 0. The decay rates of the density matrix elements γ*_nm_* are chosen as 1.0 × 10^13^ s^−1^, while the decay rates of excited states Γ_01_, Γ_12_, and Γ_0__2_ are assumed to be equal to 1.0 × 10^9^ s^−1^, 1.0 × 10^12^ s^−1^ and 0, respectively [27].

The incident laser pulse is modeled with a hyperbolic secant shape:

(11) $$E(z,t=0)=F_{0}\text{sech}\left[1.76(z/c+z_{0}/c)/\tau \right]\cos\left[\omega (z+z_{0})/c\right]$$

where the initial phase is assumed to be 0, and *F*_0_ is the peak amplitude of the input electric field. The full width at half maximum (FWHM) of the intensity profile of the pulse τ = 5 ns, unless otherwise stated. The choice of *z*_0_ ensures that the pulse penetrates negligibly into the medium at *t* = 0. In order to demonstrate the TPA-based OL behavior, we take the frequency of the incident pulse as TPA resonant frequency between the *S*_0_ and *S*_2_ states, namely, ω = ω*_S_0___S_2__*/2. The wavelength of excitation laser pulse are 876 nm for Mol-1 and Mol-2, 882 nm for Mol-3 and Mol-4, respectively. Under this condition, the molecules in the ground state *S*_0_ can absorb two photons to reach the state *S*_2_, namely, one-step resonant TPA occur (see Figure 2a). To perform the numerical simulations, the molecular density of these compounds are extracted from the experiments with *N* = 3 × 10^24^ m^−3^ [19], unless otherwise stated.

## 3. Results and Discussion

### 3.1. Temporal Evolution of the Pulse

**Figure 3:**
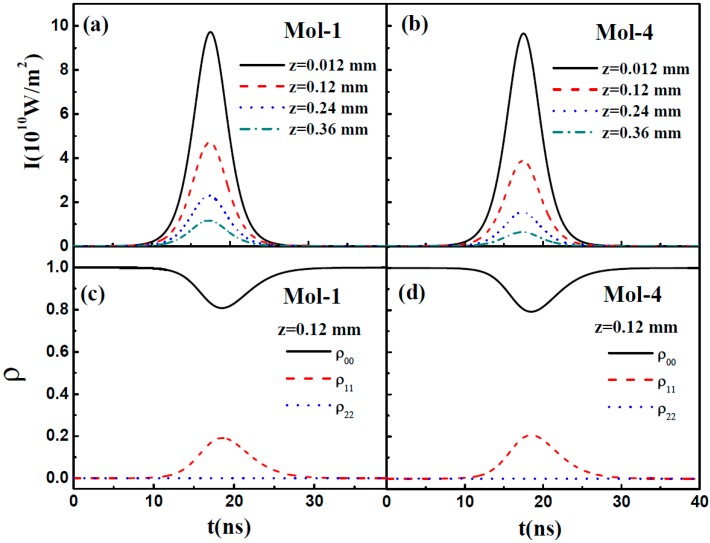
(**a**,**b**) Temporal evolution of the pulse envelope at different propagation distances and (**c**,**d**) the dynamics of the different energy level populations at the propagation distance of 0.12 mm in Mol-1 and Mol-4. (*I*_0_ = 1 × 10^11^ W/m^2^, τ = 5 ns, *N* = 3 × 10^24^ m^−3^).

To gain insight into the pulse propagation properties, Mol-1 and Mol-4 were selected as the samples to study the temporal evolution of the pulse envelope at different propagation distances (*z* = 0.012, 0.12, 0.24, 0.36 mm) and the dynamics of the populations of different energy levels at the propagation distance of 0.12 mm. The initial peak intensity *I*_0_ and the FWHM of the pulse intensity profile are set to be 1 × 10^11^ W/m^2^ and 5 ns, respectively. The molecular density of these compounds are 3 × 10^24^ m^−3^. As shown in Figure 3a,b, the pulse intensities decrease rapidly during propagation due to the energy transfer from the field to the medium through the interaction between them. Moreover, the pulse intensity in Mol-4 is much smaller than that in Mol-1 at the same propagation distance, which can be attributed to the length of the π-conjugated. Figure 3c,d demonstrate the dynamics of the different energy level populations at the propagation distance of 0.12 mm for Mol-1 and Mol-4. It shows that the particles are mainly populated on the state *S*_1_, while the state *S*_2_ is slightly populated. This is because, in nanosecond pulse regime, the populations on the *S*_2_ state will decay rapidly back to the *S*_1_ state due to the short lifetime (picosecond) of the *S*_2_ state. As a result, the change in populations on the state *S*_2_ is not obvious.

### 3.2. Optical Limiting

**Figure 4:**
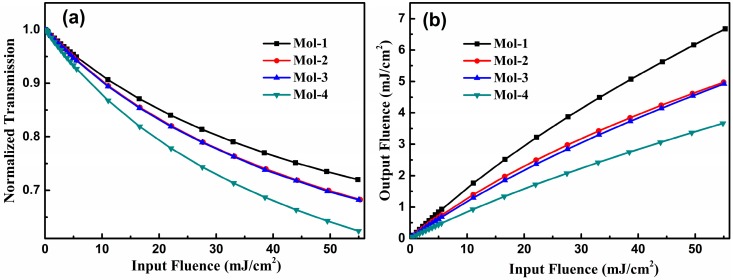
(**a**) The normalized transmission of pulse intensity versus the input fluence and (**b**) output fluence versus the input fluence of the compounds Mol-1, Mol-2, Mol-3, and Mol-4 at the same propagation distance of 0.36 mm. (τ = 5 ns, *N* = 3 × 10^24^ m^−3^).

In order to explore the mechanism by which the OL effect is achieved, the normalized transmission of pulse intensity versus the input fluence for these chromophores is plotted out in Figure 4a. The pulse width and the molecular density are the same as those in Section 3.1. This shows that transmission of the compounds decreases remarkably with the increase in input intensity, which is consistent with the experimental findings [22]. Upon excitation, molecules in the ground state *S*_0_ are excited to the *S*_2_ state by absorbing two photons; thus, the output intensity of the pulse will be strongly attenuated, leading to decreased transmission. Interestingly, compounds with an increased π-conjugated length has lower transmission owing to the enhanced TPA ability. These results show that all compounds exhibit outstanding nonlinear absorption properties. In addition, one can expect that compounds with a longer π-conjugated length possesses preferable OL performance.

So as to visualize the OL performance of the compounds, output fluence of the field as a function of the input fluence at the propagation distance of 0.36 mm is presented in Figure 4b. The OL behaviors of all chromophores are clearly demonstrated as expected, which shows a good agreement with experimental measurement [22]. Taking Mol-4 as an example, when the input fluence increased from 0 to 50 mJ/cm^2^, the output fluence changed from 0 to 3.3 mJ/cm^2^, achieving excellent OL performance. Thus, the compound can be used as a promising optical limiter candidate. Moreover, it is obvious that the molecule with a longer π-conjugated length shows stronger OL behavior. Namely, OL abilities of these chromophores decrease in the order of Mol-4 > Mol-3 > Mol-2 > Mol-1. The above results demonstrate that favourable OL performance can be achieved in this series of ladder-type tetraphenylene-cored chromophores, which is influenced by the π-conjugated length of the molecule.

**Figure 5:**
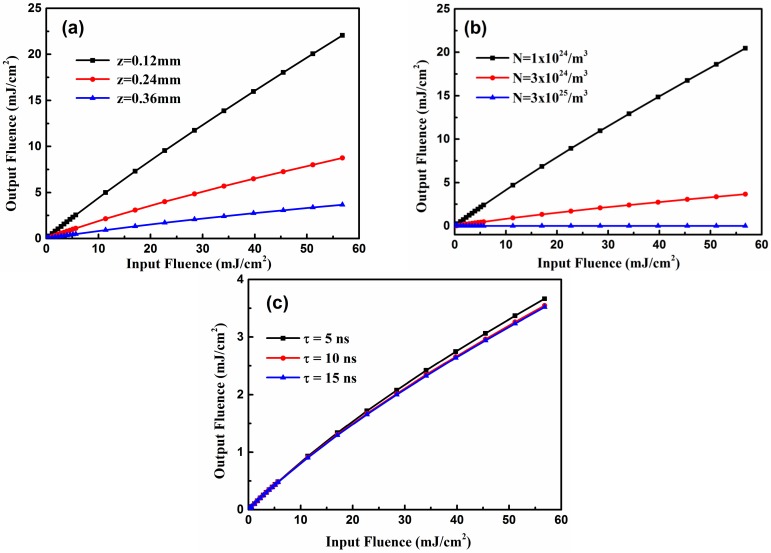
The output fluence versus input fluence of the compound Mol-4. (**a**) τ = 5 ns, *N* = 3 × 10^24^ m^−3^; (**b**) τ = 5 ns, *z* = 0.36 mm; (**c**) *N* = 3 × 10^24^ m^−3^, *z* = 0.36 mm*.*

It is interesting to explore the factors that affect the OL performance of a compound. In this section, Mol-4 is selected as the sample due to its outstanding optical limiting property according to our above investigations (see Figure 4b). In Figure 5a, the influence of sample thickness on molecular OL performance is shown with a fixed molecular density of *N* = 3 × 10^24^ m^−3^ and a pulse width of 5 ns. It is obvious that the larger the thickness, the better the OL performance. The main mechanism is that, during pulse propagation, energy transfers from field to medium through the interaction between the field and the medium; thus, OL performance is enhanced as the propagation distance increases. Figure 5b displays the OL performances of Mol-4 with different molecular densities (*N* = 1 × 10^24^, 3 × 10^24^, 3 × 10^25^ m^−3^) at a distance of *z* = 0.36 mm with a fixed pulse width of 5 ns. Intuitively, a larger concentration results in stronger optical limiting behavior at the same distance since more particles can participate in the interaction between the medium and field. The above results indicate that increasing both the length and the concentration of the absorber will cause the interaction between the field and the medium to become stronger and thus result in enhanced OL performance of the medium.

On the basis of the results that parameters of the medium can influence the OL performance of the compounds, we explored whether the parameter of the laser pulse could affect the nonlinear optical properties of the medium. To elucidate the effect of pulse duration on OL behavior, the output fluence versus input fluence curves with different pulse widths (τ = 5, 10, 15 ns) at the same propagation distance of 0.36 mm is shown in Figure 5c. In comparison with shorter pulses, longer pulses induce stronger OL performance. By the mechanism of the TPA process, when the medium interacts with a pulse with a wider duration, the interaction between the molecule and the laser pulse stays longer, and populations in state *S*_0_ have larger probabilities of being excited to state *S*_2_. As a result, longer pulses obtain OL enhancement.

From the above numerical results, it is noted that one can modulate the optical limiting property of the absorbor by changing either the parameters of the media, such as the thickness and the concentration, or the parameter of the laser pulse, such as the pulse width. These conclusions are consisitent with previous studies where 4,4′-bis(diphenylamino) stilbene and 4,4′-bis(dimethylamino) stilbene were used as optical limiting materials [29,30].

### 3.3. Dynamical TPA Cross Section

By fitting the input–output peak intensity curves within the optical limiting region of the compounds, we further obtain OPA coefficient α, TPA coefficient β, and TPA cross section σ_tp_ at different propagation distances for Mol-1, Mol-2, Mol-3, and Mol-4 with a pulse width of 5 ns and a molecular density of 3 × 10^24^ m^−3^. The corresponding values are collected in Table 2. The dynamical TPA coefficients are obtained as 3.64 × 10^−8^ m/W, 4.01 × 10^−8^ m/W, 4.12 × 10^−8^ m/W, and 5.09 × 10^−8^ m/W for Mol-1, Mol-2, Mol-3, and Mol-4, respectively, for a propagation distance of 0.36 mm and a pulse duration of 5 ns. The corresponding dynamical values of the TPA cross sections are 2.75 × 10^7^ GM, 3.07 × 10^7^ GM, 3.10 × 10^7^ GM, and 3.85 × 10^7^ GM for Mol-1, Mol-2, Mol-3, and Mol-4, respectively, showing an enhanced TPA cross section for the compound with a longer π-conjugated length. Our calculations further validate the fact that a longer π-conjugated length induces a larger TPA cross section, even though this universal rule has been testified with respect to different molecules both from experimental observations and theoretical simulations [31,32]. It is noted that the theoretical results for different molecules show the same trends as the experimental measurement [22], but the calculated values are of several orders of magnitude larger than the experimental values. The discrepancy between the simulation and experiment may mainly result from the off-resonant condition in measurement, while our simulation is on the resonant TPA case. Moreover, the experiment was performed using a laser pulse with a 160 fs duration, while, in our calculations, laser pulses in the nanosecond time domain were employed.

Taking the propagation into consideration, dynamical TPA cross sections of Mol-4 where pulse width τ = 5 ns are 3.57 × 10^7^ GM, 3.77 × 10^7^ GM, and 3.85 × 10^7^ GM for the propagation distance of 0.12 mm, 0.24 mm, and 0.36 mm, respectively. It is evident that a dynamical TPA cross section depends crucially on the thickness of the medium, which is remarkably consistent with previous studies [24,33,34]. Thus, one should consider this factor when one compares TPA cross sections in different measurements.

## 4. Conclusions

The dynamical processes of nanosecond laser pulses propagated in the newly-synthesized ladder-type chromophores containing oligo-*p*-phenylene moiety were simulated, and the TPA-based OL performances of these compounds were theoretically studied. The results show that all of the studied compounds exhibit strong nonlinear optical properties, indicating them as promising candidates for optical limiters. Moreover, the increase in the π-conjugated length of this series of compounds results in remarkable OL performance and an enhanced dynamical TPA cross section. Our results agree well with other experimental measurements. Most importantly, the factors that can influence the OL performance of the molecules were investigated, and it was found that the thickness and concentration of the absorber as well as the pulse duration play key roles in the OL performance of these molecules. It is suggested that the nonlinear optical absorption enhancement can be achieved by modulating the parameters of the absorber.

## Figures and Tables

**Table 1:** The excitation energy *E_n_*_0_ (eV), the corresponding wavelength λ*_n_*_0_ (nm), the oscillator strength δ_op_ (a.u.) and the dipole moment μ*_mn_* (a.u.) of the first five excited states of Mol-1, Mol-2, Mol-3, and Mol-4 in gas phase.

Mol.	Excited State *n*	*E_n_*_0_	λ*_n_*_0_	δ_op_	μ_0*n*_	μ_1*n*_	μ_2*n*_	μ_3*n*_	μ_4*n*_	μ_5*n*_
Mol-1	1	2.51	494	3.50	7.54	0.40	-	-	-	-
	2	2.83	438	0.01	0.45	14.38	0.90	-	-	-
	3	3.10	400	0.09	1.12	4.32	0.48	0.02	-	-
	4	3.23	383	0.84	3.25	0.66	18.09	5.13	0.66	-
	5	3.33	372	0.07	0.97	0.94	3.68	12.19	0.04	0.33
Mol-2	1	2.53	490	4.10	8.12	0.15	-	-	-	-
	2	2.83	438	0.00	0.07	15.97	0.19	-	-	-
	3	2.98	416	0.00	0.03	6.01	0.28	0.74	-	-
	4	3.12	398	0.80	3.62	0.01	22.36	11.47	0.10	-
	5	3.24	382	0.00	0.17	0.11	3.33	10.56	0.02	0.19
Mol-3	1	2.53	490	4.45	8.46	0.30	-	-	-	-
	2	2.81	441	0.04	0.77	15.49	1.27	-	-	-
	3	2.90	428	0.08	1.10	5.40	0.25	0.22	-	-
	4	3.04	407	0.97	3.61	0.23	23.08	18.47	0.71	-
	5	3.16	392	0.00	1.26	1.38	7.53	8.88	0.25	0.55
Mol-4	1	2.55	486	5.10	9.03	0.06	-	-	-	-
	2	2.81	441	0.00	0.07	15.63	0.62	-	-	-
	3	2.86	434	0.00	0.13	6.79	0.95	0.14	-	-
	4	2.99	415	0.92	3.54	0.20	16.15	30.72	1.59	-
	5	3.11	399	0.00	0.16	0.24	12.80	2.96	0.59	0.70

**Table 2:** OPA coefficient α (10^3^/m), TPA coefficient β (10^−8^ m/W), and TPA cross section σ_tp_ (10^7^ GM) (1 GM = 1 × 10^−50^ cm^4^s/photon) at different propagation distances of *z* (mm) for Mol-1, Mol-2, Mol-3, and Mol-4 with a pulse width of τ *=* 5 ns and the molecular density of *N* = 3 × 10^24^ m^−3^.

Mol.	Mol-1			Mol-2			Mol-3			Mol-4		
*z*	α	β	σ_tp_	α	β	σ_tp_	α	β	σ_tp_	α	β	σ_tp_
0.12	4.65	3.39	2.56	5.18	3.70	2.83	5.46	3.81	2.86	6.38	4.72	3.57
0.24	4.81	3.57	2.70	5.37	3.93	3.00	5.66	4.04	3.03	6.61	4.99	3.77
0.36	4.87	3.64	2.75	5.43	4.01	3.07	5.72	4.12	3.10	6.68	5.09	3.85

## References

[1] Naseema K., Manjunatha K.B., Sujith K.V., Umesh G., Kalluraya B., Rao V. (2012). Third order optical nonlinearity and optical limiting studies of propane hydrazides. Opt. Mater..

[2] Yao C.B., Zhang Y.D., Chen D.T., Yin H.T., Yu C.Q., Li J., Yuan P. (2013). Study of all-optical switching and optical limiting properties in phenoxy-phthalocyanines liquid. Opt. Laser Technol..

[3] Zhu J.H., Li Y.X., Chen Y., Wang J., Zhang B., Zhang J.J., Blau W.J. (2011). Graphene oxide covalently functionalized with zinc phthalocyanine for broadband optical limiting. Carbon.

[4] Feng M., Zhan H., Chen Y. (2010). Nonlinear optical and optical limiting properties of graphene families. Appl. Phys. Lett..

[5] Ma H., Leng J.C., Liu M., Zhao L.N., Jiao Y. (2015). Two-photon absorption and optical limiting of a fluorenyl-based chromophore with femtosecond laser pulse. Opt. Commun..

[6] Xu H., Song Y.L., Meng X.R., Hou H.W., Tang M.S., Fan Y.T. (2009). Strong optical limiting effects of two Ag(I)-bridged metal-organic polymers. Chem. Phys..

[7] Liu J.C., Wang C.K., Gel’mukhanov1 F. (2007). Optical limiting of short laser pulses. Phys. Rev. A.

[8] Tutt L.W., Boggess T.F. (1993). A review of optical limiting mechanisms and devices using organics, fullerenes, semiconductors and other materials. Prog. Quantum Electron..

[9] Chang Y.C., Chiou A.E., Khoshnevissan M. (1992). Linear and two-photon absorptions of Si–Ge strained-layer superlattices. J. Appl. Phys..

[10] He G.S., Reinhardt B.A., Bhatt J.C., Dillard A.G., Xu G.C., Prasad P.N. (1995). Two-photon absorption and optical-limiting properties of novel organic compounds. Opt. Lett..

[11] Price R.S., Dubinina G., Wicks G., Drobizhev M., Rebane A., Schanze K.S. (2015). Polymer monoliths containing two-photon absorbing phenylenevinylene platinum(II) acetylide chromophores for optical power limiting. ACS Appl. Mater. Interfaces.

[12] Varnavski O., Yan X.Z., Mongin O., Desce M.B., Goodson T. (2007). Strongly interacting organic conjugated dendrimers with enhanced two-photon absorption. J. Phys. Chem. C.

[13] Tian Y.Q., Chen C.Y., Cheng Y.J., Young A.C., Tucker N.M., Jen A.K.Y. (2007). Hydrophobic chromophores in aqueous micellar solution showing large two-photon absorption cross sections. Adv. Funct. Mater..

[14] He G.S., Tan L.S., Zheng Q.D., Prasad P.N. (2008). Multiphoton absorbing materials: Molecular designs, characterizations, and applications. Chem. Rev..

[15] Belfield K.D., Bondar M.V., Hernandez F.E., Przhonska O.V. (2008). Photophysical characterization, two-photon absorption and optical power limiting of two fluorenylperylene diimides. J. Phys. Chem. C.

[16] Zheng Q.D., He G.S., Prasad P.N. (2005). π-conjugated dendritic nanosized chromophore with enhanced two-photon absorption. Chem. Mater..

[17] Li C.W., Yang K., Feng Y., Su X.Y., Yang J.Y., Jin X., Shui M., Wang Y.X., Zhang X.R., Song Y.L. (2009). Investigation of two-photon absorption induced excited state absorption in a fluorenyl-based chromophore. J. Phys. Chem. B.

[18] Reinhardt B.A., Brott L.L., Clarson S.J., Dillard A.G., Bhatt J.C., Kannan R., Yuan L., He G.S., Prasad P.N. (1998). Highly active two-photon dyes: Design, synthesis, and characterization toward application. Chem. Mater..

[19] Jacob J., Sax S., Gaal M., List E.J.W., Grimsdale A.C., Müllen K. (2005). Afully aryl-substituted poly(Ladder-type pentaphenylene): A remarkable stable blue-light emitting polymer. Macromolecules.

[20] Laquai F., Mishra A.K., Ribas M.R., Petrozza A., Jacob J., Akcelrud L., Müllen K., Friend R.H., Wegner G. (2007). Photophysical properties of a series of poly(ladder-type pentaphenylene)s. Adv. Funct. Mater..

[21] Jacob J., Sax S., Piok T., List E.J.W., Grimsdale A.C., Müllen K. (2004). Ladder-type pentaphenylenes and their polymers: Efficient blue-light emitters and electron-accepting materials via a common intermediate. J. Am. Chem. Soc..

[22] Zheng Q.D., Gupta S.K., He G.S., Tan L.-S., Prasad P.N. (2008). Synthesis, characterization, two-photon absorption, and optical limiting properties of ladder-type Oligo-*p*-phenylene-Cored Chromophores. Adv. Funct. Mater..

[23] Liu J.C., Felicíssimo V.C., Guimarães F.F., Wang C.K., Gel’mukhanov F. (2008). Coherent control of population and pulse propagation beyond the rotating wave approximation. J. Phys. B At. Mol. Opt. Phys..

[24] Baev A., Gel’mukhanov F., Macák P., Luo Y., Ågren H. (2002). General theory for pulse propagation in two-photon active media. J. Chem. Phys..

[25] Ziolkowski R.W., Arnold J.M., Gobny D.M. (1995). Ultrafast pulse interactions with two-level atoms. Phys. Rev. A.

[26] Dalton A Molecular Electronic Structure Program. http://www.kjemi.uio.no/software/dalton/dalton.html.

[27] Gavrilyuk S., Polyutov S., Jha P.C., Rinkevicius Z., Ågren H., Gel’mukhanov F. (2007). Many-photon dynamics of photobleaching. J. Phys. Chem. A.

[28] Sun Y.P., Liu J.C., Wang C.K. (2009). Effect of time-dependent ionization on dynamical two-photon absorption cross sections of molecular media. Acta Opt. Sin..

[29] Wang C.K., Zhao P., Miao Q., Sun Y.P., Zhou Y. (2010). Optical limiting and dynamical two-photon absorption of organic compounds for a nanosecond pulse. J. Phys. B At. Mol. Opt. Phys..

[30] Zhou Y., Miao Q., Sun Y.P., Gel’mukhanov F., Wang C.K. (2011). Solvent effect on dynamical TPA and optical limiting of BDMAS molecular media for nanosecond and femtosecond laser pulses. J. Phys. B At. Mol. Opt. Phys..

[31] Cho B.R., Son K.H., Lee S.H., Song Y.-S., Lee Y.-K., Jeon S.-J., Choi J.H., Lee H., Cho M. (2001). Two photon absorption properties of 1,3,5-Tricyano-2,4,6-tris(styryl)benzene derivatives. J. Am. Chem. Soc..

[32] Han G.C., Zhao K., Liu P.W., Zhang L.L. (2012). Influence of rotational isomerism on two-photon absorption properties of FTC chromophores. Chin. Phys. B.

[33] Ma H., Leng J.C., Liu M., Zhao L.N., Jiao Y. (2015). Theoretical investigation of two-photon absorption properties and optical limiting behavior of two symmetrical fluorene derivatives. J. Nonlinear Opt. Phys. Mater..

[34] Wang C.K., Liu J.C., Zhao K., Sun Y.P., Luo Y. (2007). Breakdown of optical power limiting and dynamical two-photon absorption for femtosecond laser pulses in molecular medium. J. Opt. Soc. Am. B.

